# Prognostic impact of methylation-related gene mutations in elderly acute myeloid leukemia: a real-world retrospective analysis

**DOI:** 10.3389/fmed.2025.1594784

**Published:** 2025-05-13

**Authors:** Yi Chen, Zhengjun Wu, Yanxin Chen, Zhengyang Wang, Ruyu Cai, Yong Wu, Jing Zheng

**Affiliations:** ^1^Fujian Provincial Key Laboratory on Hematology, Fujian Institute of Hematology, Fujian Medical University Union Hospital, Fuzhou, China; ^2^School of Basic Medical Science, Fujian Medical University, Fuzhou, China

**Keywords:** elderly, acute myeloid leukemia, methylation, mutation, prognosis

## Abstract

**Objective:**

This study aimed to evaluate the prognostic impact of methylation-related gene mutations in older patients with acute myeloid leukemia (AML).

**Methods:**

We conducted a retrospective analysis of clinical characteristics in 645 patients aged ≥ 60 years diagnosed with AML at Fujian Medical University Union Hospital between July 2016 and December 2024.

**Results:**

Methylation-related gene mutations—specifically *DNMT3A*, *TET2*, *IDH1*, and *IDH2*—were identified in 24.0%, 22.5%, 9.1%, and 13.8% of cases, respectively. Patients with single mutations in *DNMT3A* or *TET2* exhibited similar long-term survival outcomes compared to those without these mutations, with median survival times of 25.2 and 22.3 months, respectively (*p* = 0.9639). However, patients with concurrent *DNMT3A* and *TET2* mutations demonstrated the poorest treatment response and prognosis, achieving a complete remission (CR) rate of 35.5% and a median survival of only 6.2 months. In contrast, patients with *IDH1*/*IDH2* mutations responded better to treatment, achieving a CR rate of 69.6% and a median survival of 34.7 months. Treatment regimens combining azacitidine and venetoclax did not provide additional improvement in treatment response for patients with methylation-related gene mutations compared to intensive chemotherapy (IC).

**Conclusion:**

Concurrent mutations in *DNMT3A* and *TET2* were associated with significantly poorer treatment response and survival outcomes. These common methylation-related gene mutations did not influence the choice between IC and azacitidine plus venetoclax combination therapy in elderly AML patients.

## 1 Introduction

Acute myeloid leukemia (AML) is one of the most common subtypes of leukemia in the elderly population (1, 2). Regrettably, the survival rates for this age group remain alarmingly low, largely due to the high prevalence of adverse cytogenetic prognostic factors (3–5), with five-year survival rates reported to be below 10% (2, 6, 7). Current treatment strategies for elderly AML patients are tailored based on factors including age, physical fitness, and the presence of adverse genetic prognostic markers (8, 9). Approximately 40% to 60% of physically fit patients treated with standard intensive chemotherapy (IC) regimens achieve complete remission (CR) (10). For patients unsuitable for IC, a combination therapy of hypomethylating agents (HMAs) and venetoclax is recommended as the frontline treatment (9, 11).

Methylation alterations play a pivotal role in the pathogenesis of cancer by modulating gene expression through changes in chromatin structure and the accessibility of transcription factors to DNA (12–14). In leukemia, aberrant methylation patterns, including hypermethylation of tumor suppressor genes or hypomethylation of oncogenes, can disrupt normal gene expression, thereby contributing to disease development (15, 16). Several methylation-related gene mutations have been identified in AML, with *DNMT3A*, *TET2*, *IDH1*, and *IDH2* being the most frequently affected (17–20). However, the prognostic implications of these gene mutations in elderly AML patients, as well as their potential impact on the efficacy of IC or combined HMA and venetoclax regimens, are not well elucidated and necessitate further study.

In light of these considerations, this study aims to evaluate the impact of prevalent methylation-related gene mutations on the prognosis of elderly AML patients. Additionally, we seek to determine whether these mutations should influence the decision-making process when selecting between IC and combination treatment regimens incorporating HMAs. This research is critically important for refining therapeutic strategies tailored to the practical needs of elderly AML patients.

## 2 Methods

### 2.1 Study population

This retrospective study included a cohort of 645 elderly patients (aged 60 years and above) who were diagnosed with AML, spanning from July 2016 to December 2024 at the Fujian Medical University Union Hospital. Patients with available genetic mutation profiles were included in this study, whereas those lacking documented molecular analysis data were systematically excluded. A retrospective analysis of patient medical records enabled systematic compilation of multidimensional clinical datasets encompassing, including hematologic parameters and biochemical profiles, genetic aberrations, therapeutic protocols, objective treatment responses and long-term survival. This study was conducted in accordance with the principles of the Declaration of Helsinki. This retrospective cohort study utilizing anonymized clinical data was classified as non-interventional research under Declaration of Helsinki. In accordance with ICH-GCP guidelines regarding secondary data use, the requirement for written informed consent was formally waived by the Institutional Review Board of Fujian Medical University Union Hospital (approval no. 2024KY278), as the research posed no additional risks and preserved participant confidentiality.

### 2.2 Definition of AML and genetic mutations

The diagnosis of AML was established in accordance with the criteria provided in the 2016 revision of the World Health Organization (WHO) Classification of Tumours of Hematopoietic and Lymphoid Tissues, specifically pertaining to myeloid neoplasms and acute leukemia (21). Secondary AML (s-AML) was defined as AML that arose from pre-existing myeloproliferative disorders, such as myelodysplastic syndromes (MDS), myeloproliferative neoplasms (MPN), and chronic myelomonocytic leukemia (CMML). Patients with the blast phase of chronic myeloid leukemia (CML) were not included in this study. Therapy-related AML (t-AML) was identified as AML that developed following prior chemotherapy or radiation therapy for another malignancy. The chromosomal karyotype analysis was performed using R-banding technique in our center, and a complex karyotype was defined as the presence of three or more chromosomal aberrations. Gene mutations analysis were mainly conducted through targeted next-generation sequencing (NGS) using a custom-designed myeloid-focused gene panel (Kangsheng Global Medical Technology, Beijing) covering clinically actionable genes. Sequencing was performed on the Illumina NovaSeq X Plus platform, and the bioinformatic processing utilized an automated pipeline for hematologic neoplasm genomic reporting. The specific gene regions analyzed for mutation detection are summarized in Supplementary Table 1.

### 2.3 Treatment protocols

In this study, the “IC” regimen denotes the standard 3 + 7 protocol. Following the achievement of complete remission (CR) or complete remission with incomplete recovery (CRi) after IC treatment, intermediate or high-dose cytarabine was used as a consolidation therapy. Hypomethylating agent (HMA)-based therapies were administered either as monotherapy or in combination with low-dose chemotherapy regimens. For patients who achieved CR with HMA-based treatments, the same regimen was continued for consolidation. The azacitidine-venetoclax regimen utilized the standard dosage and administration. For patients who achieved CR with this combination, the concurrent administration of AZA and VEN was sustained until disease progression or intolerable adverse effects arose. A detailed summary of the initial treatment protocols for these patients, including the specific therapeutic approaches utilized, is provided in Supplementary Table 2.

### 2.4 Definition of response and outcomes

The effectiveness of therapeutic interventions was assessed in accordance with the 2022 edition (5th) of the European LeukemiaNet (ELN) response criteria for AML (8). Complete blood count measurements were obtained at the commencement of each treatment cycle, and bone marrow aspirations were conducted at intervals of one or two cycles to evaluate the therapeutic response. The overall response rate (ORR) was determined by combining the rates of CR, CR with incomplete hematologic recovery (CRi), and partial remission (PR). To identify minimal residual disease (MRD) in AML, an 8-color flow cytometric protocol was utilized at our center, employing the FACSCanto flow cytometer from BD company, United States. The protocol was designed with a lower limit of detection set at 10^−4^. Relapse was defined as the return of leukemic cells in the bone marrow or peripheral blood after achieving CR/CRi, indicated by blast cell percentages greater than 5%, or the appearance of extramedullary lesions. The duration of remission (DOR) was calculated as the time span from the achievement of CR/CRi or PR to the occurrence of relapse or disease progression. Overall survival (OS) was defined as the time period from the date of diagnosis to the date of death or the last date of follow-up.

### 2.5 Statistical analysis

The statistical evaluation of differences between patient subgroups was carried out using the chi-square test for categorical variables, the t-test for continuous data that were normally distributed, or nonparametric tests for data that did not conform to the assumptions of parametric tests. The Kaplan–Meier method was used for univariate analysis to assess the prognostic impact of methylation-related gene mutations compared to wild-type and other clinical and laboratory indicators. Log-rank tests were conducted to compare differences between groups. All statistical computations and graphical illustrations were performed with GraphPad Prism 9.5 (GraphPad Software, San Diego, CA, United States). Statistical significance was established at a *p*-value of less than 0.05.

## 3 Results

### 3.1 Clinical characteristics

The study cohort comprised 645 individuals, with a male-to-female ratio of approximately 1.70:1, including 406 males (62.9%) and 239 females (37.1%). The age of the patients ranged from 60 to 98 years, with a median age of 67 years. Among these patients, 511 (79.2%) had *de novo* AML, 97 (15.0%) had s-AML, and 37 (5.7%) had t-AML. Myelodysplastic syndromes (MDS) were the most frequent precursors of s-AML, representing 73.2% of cases.

**Figure 1:**
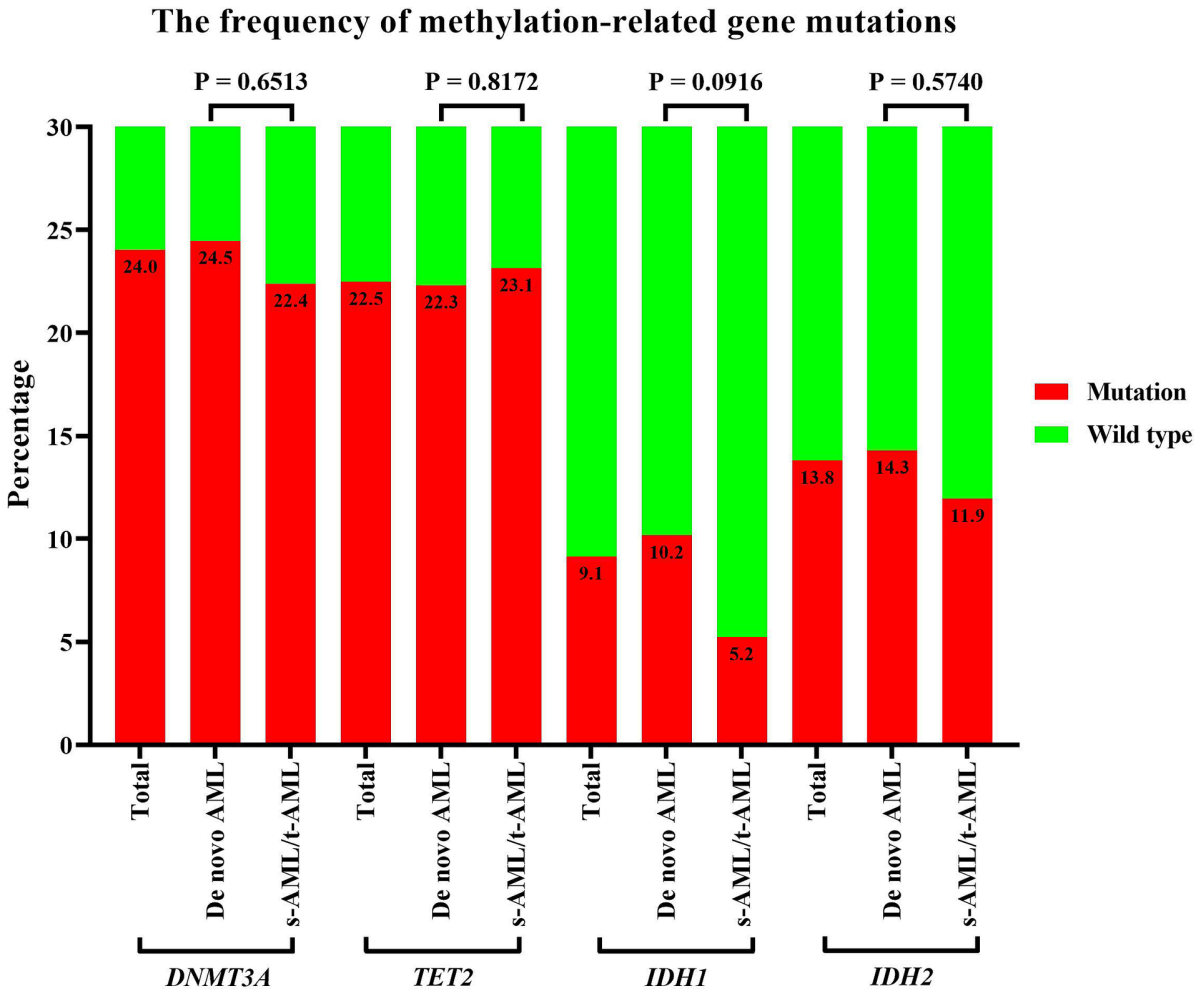
Incidence of methylation-related gene mutations in older patients with AML. Incidence of methylation-related gene (*DNMT3A*, *TET2*, *IDH1/2*) mutations stratified by disease subtype: *de novo* AML vs. therapy-related/secondary AML. Statistical comparisons used Fisher’s exact test, with *p*-values: *DNMT3A* = 0.651, *TET2* = 0.817, *IDH1* = 0.092, *IDH2* = 0.574. AML, acute myeloid leukemia.

Cytogenetic analysis results were available for a subset of patients with 40.7% displaying abnormal chromosome karyotypes. Specific abnormalities included deletions of chromosome 5 or 5q- in 6.8%, chromosome 7- in 3.3%, chromosome 17- in 1.9%, chromosome +8 in 10.3%, and complex karyotypes in 17.5% of cases. As depicted in Figure 1, methylation-related gene mutations, including *DNMT3A*, *TET2*, *IDH1*, and *IDH2*, were observed in 24.0%, 22.5%, 9.1%, and 13.8% of patients, respectively. The incidence of *IDH1* gene mutations was more occurred in *De novo* AML compared to s-AML/t-AML, and there was no significant difference in the occurrence rates of *DNMT3A*, *TET2*, and *IDH2* gene mutations between these two AML subtypes. *FLT3* gene mutations were found in 21.2% of patients, and other commonly detected mutations included those in *NPM1*, *CEBPA*, *TP53*, *ASXL1*, and *RUNX1*, with positive rates of 21.4%, 12.9%, 14.1%, 17.1%, and 13.8%, respectively. Based on genetic risk classification, the adverse group was the most prevalent, accounting for 55.3%, while the favorable and intermediate groups represented 18.0% and 26.7% of the patients, respectively. A comprehensive summary of the patients’ characteristics is provided in Table 1.

**Table 1:** Baseline clinical and genetic characteristics of all patients.

Parameters	Results
Total, *n*	645
Median age, year (range)	67 (60–98)
Male sex, *n* (%)	406 (62.9)
Leukemia subtype, *n* (%)	
De novo AML	511 (79.2)
s-AML	97 (15.6)
MDS	71 (11.3)
t-AML	37 (4.6)
Median WBC, 10^9^/L (range)	11.50 (0.03–382.40)
Median HB, g/L (range)	71 (25–145)
Median PLT, 10^9^/L (range)	44 (1–767)
Median serum LDH, IU/L (range)	340 (77–6,432)
Median BM blast, % (range)	59.0 (11.5–99.0)
Chromosome karyotype, *n* (%)	
Normal	305 (59.3)
Abnormal	209 (40.7)
−5 or 5q-	35 (6.8)
−7	17 (3.3)
−17	10 (1.9)
+8	53 (10.3)
Complex	90 (17.5)
Unavailable^1^	131 (20.3)
Fusion genes, *n* (%)	
Negative	489 (82.7)
Positive	102 (17.3)
*AML1/ETO*	19 (3.2)
*CBFβ/MYH11*	5 (0.8)
*MLL1*	31 (5.2)
*HOX11*	13 (2.2)
*ETV1*	13 (2.2)
Unavailable^1^	54 (9.1)
Gene mutations	
Negative	57 (8.8)
Positive	588 (91.2)
*FLT3*	137 (21.2)
*NPM1*	138 (21.4)
*CEBPA*	83 (12.9)
*TP53*	93 (14.1)
*ASXL1*	110 (17.1)
*RUNX1*	89 (13.8)
Risk classification by genetics, *n* (%)	
Favorable	114 (18.0)
Intermediate	169 (26.7)
Adverse	350 (55.3)
Unavailable^1^	12 (1.9)

MDS, myelodysplastic syndrome; AML, acute myeloid leukemia; MPN, myeloproliferative neoplasms; CMML, chronic myelomonocytic leukemia; WBC, white blood cell; HB, hemoglobin; PLT, platelet; LDH, lactate dehydrogenase; BM, bone marrow. ^1^Due to various reasons, including bone marrow sample clotting, testing conducted at external hospitals, and the absence of specific tests, some patients were unable to obtain results for karyotype analysis and fusion genes, resulting the risk classification by genetics was unavailable.

### 3.2 Treatment strategies and outcomes

Of the 645 patients in the study cohort, 585 (90.7%) received further anti-leukemia treatment following diagnosis. The treatments included 240 patients (41.0%) undergoing IC, 43 patients (7.4%) receiving HMAs as monotherapy, 91 patients (15.6%) treated with a combination of HMAs and low-dose chemotherapy, 183 patients (31.3%) administered AZA in combination with VEN, and 28 patients (4.8%) managed with other therapeutic regimens. The baseline characteristics of patients receiving various treatment regimens were summarized in Table 2. From the table, it could be observed that patients in the IC group were relatively younger compared to those in other groups. Additionally, the proportions of s-AML/t-AML and adverse cytogenetics in the IC group were significant lower than those in the other treatment groups.

**Table 2:** Baseline characteristics of patients by treatment regimen.

Parameters	IC (*N* = 240)	HMAs (*N* = 43)	HMAs + chemo (*N* = 91)	AZA + VEN (*N* = 183)	Others (*N* = 28)	*p*-value
Median age, year (range)	64 (60–77)	70 (61–82)	67 (60–80)	70 (60–91)	67 (60–80)	<0.0001
Male, *n* (%)	152 (63.3)	31 (72.1)	57 (62.6)	110 (60.1)	18 (64.3)	<0.0001
s-AML/t-AML, *n* (%)	21 (8.8)	19 (44.2)	34 (37.4)	41 (22.4)	7 (25.0)	<0.0001
Median WBC, 10^9^/L (range)	24.86 (0.42–312.20)	3.35 (0.46–138.00)	4.36 (0.19–324.30)	6.50 (0.03–328.40)	2.08 (0.57–200.80)	<0.0001
Median HB, g/L (range)	77 (29–145)	76 (46–119)	69 (25–144)	68 (28–127)	67 (46–108)	0.0013
Median PLT, 10^9^/L (range)	46 (1–360)	48 (5–340)	45 (2–557)	42 (2–767)	43 (11–367)	0.5593
Median LDH, IU/L (range)	408 (77–3,657)	240 (103–1,683)	274 (113–2,714)	312 (115–6,432)	302 (143–1,532)	0.0288
Median BM blast, % (range)	71.0 (20.0–99.0)	34.0 (17.0–93.0)	44.5 (11.5–87.0)	50.5 (12.0–98.5)	70.0 (20.0–98.0)	<0.0001
Risk classification by genetics^1^, *n* (%)						
Favorable	65 (27.2)	7 (16.7)	14 (15.4)	22 (12.1)	0 (0)	<0.0001
Intermediate	72 (30.1)	11 (26.2)	22 (24.2)	41 (22.5)	10 (35.7)	0.2233
Adverse	102 (42.7)	24 (57.1)	55 (60.4)	119 (65.4)	18 (64.3)	<0.0001

IC, intensive chemotherapy; HMA, hypomethylating agent; AZA, azacitidine; VEN, venetoclax; AML, acute myeloid leukemia; WBC, white blood cell; HB, hemoglobin; PLT, platelet; LDH, lactate dehydrogenase; BM, bone marrow. ^1^Due to various reasons, including bone marrow sample clotting, testing conducted at external hospitals, and the absence of specific tests, some patients were unable to obtain results for karyotype analysis and fusion genes, resulting the risk classification by genetics was unavailable.

**Figure 2:**
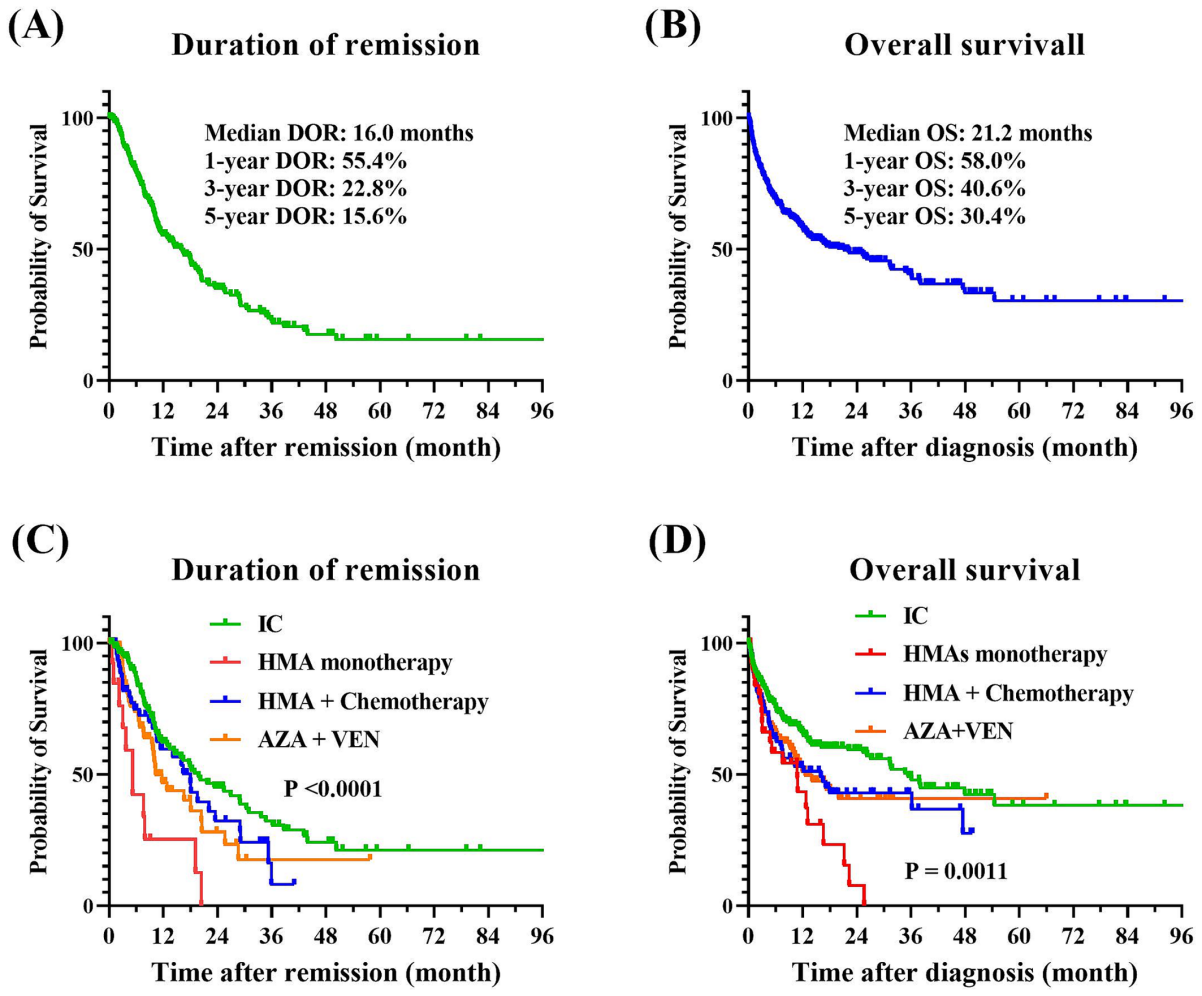
Treatment outcomes of older patients with AML. **(A)** DOR for the entire cohort. **(B)** OS for the entire cohort. **(C)** Comparative DOR among various treatment groups (*p* < 0.0001). **(D)** Comparative OS among various treatment groups (*p* = 0.0011). The Kaplan–Meier method was employed to perform univariate analysis of the prognostic impact of treatment regimens, and the log-rank tests were utilized to assess the differences between the groups. AML, acute myeloid leukemia; DOR, duration of remission; OS, overall survival.

Among the 585 patients who received anti-leukemia therapy, 54 (9.2%) were lost to follow-up, and 66 (11.3%) died during the induction phase. As illustrated in Figure 2, the median DOR was 16.0 months, with 1-year, 3-year, and 5-year DOR rates of 55.4, 22.8, and 15.6%, respectively. The median OS was 21.2 months, with corresponding 1-year, 3-year, and 5-year OS rates of 58.0%, 40.6%, and 30.4%. Across the entire cohort, the induction treatment CR rate was 51.2%, the PR rate was 13.3%, the ORR rate was 64.5%, and the MRD-negative rate was 30.1%. Obviously, patients treated with IC showed the most favorable treatment response and long-term outcomes, with a CR rate of 58.7% and median DOR and OS of 19.8 months and 35.4 months, respectively, and a 5-year OS of 38.2%. The AZA and VEN combination therapy also resulted in a satisfactory response, with a CR rate of 56.3%, which was comparable to the IC group (*p* = 0.7331). HMA monotherapy had the least favorable outcomes, with a CR rate of 17.6% and median DOR and OS of only 5.2 months and 10.8 months, respectively. In contrast, the combination of HMAs with low-dose chemotherapy was more effective, with a CR rate of 39.7% and median DOR and OS of 18.0 and 16.0 months, respectively. The highest proportions of MRD-negative responses were observed in patients treated with IC and AZA + VEN, at 37.5% and 32.0%, respectively, while all other treatment protocols resulted in MRD-negative rates of less than 20%. The comprehensive treatment response and prognosis for patients undergoing various therapeutic protocols were summarized in Table 3.

**Table 3:** Outcomes of patients treated with various regimens.

Parameters	IC (*N* = 240)	HMAs (*N* = 43)	HMAs + chemo (*N* = 91)	AZA + VEN (*N* = 183)	Others (*N* = 28)	*p*-value
Treatment response						
CR/CRi, *n* (%)	122 (58.7)	6 (17.6)	29 (39.7)	72 (56.3)	9 (40.9)	<0.0001
PR, *n* (%)	20 (9.6)	7 (20.6)	17 (23.3)	15 (11.7)	3 (13.6)	0.0212
ORR, *n* (%)	142 (68.3)	13 (38.2)	46 (63.0)	87 (68.0)	12 (54.5)	0.0102
MRD-negative, *n* (%)^1^	78 (37.5)	3 (8.8)	14 (19.2)	41 (32.0)	4 (18.1)	0.0010
Mortality, *n* (%)	22 (9.2)	2 (5.9)	11 (12.1)	26 (14.2)	5 (17.9)	0.2150
Follow-up missed, *n* (%)	10 (4.2)	7 (20.6)	7 (7.7)	29 (15.8)	1 (3.6)	0.0003
Long-term survival						
Median DOR, month	19.8	5.2	18.0	10.3	11.0	<0.0001
3-year DOR, (%)	32.3	0	8.1	17.6	0	
Median OS, month	35.4	10.8	16.0	12.6	25.2	0.0011
3-year OS, (%)	49.2	0	42.9	40.9	0	

IC, intensive chemotherapy; HMA, hypomethylating agent; AZA, azacitidine; VEN, venetoclax; CR, complete remission; CRi, complete remission with incomplete recovery; PR, partial remission; ORR, overall remission; MRD, minimal residual disease; DOR, duration of remission; OS, overall survival. ^1^Due to the unavailability of MRD testing technology at our hospital during their hospitalization, a small number of early-stage patients did not have corresponding MRD results.

### 3.3 Prognostic impact of methylation-related gene mutations

**Figure 3:**
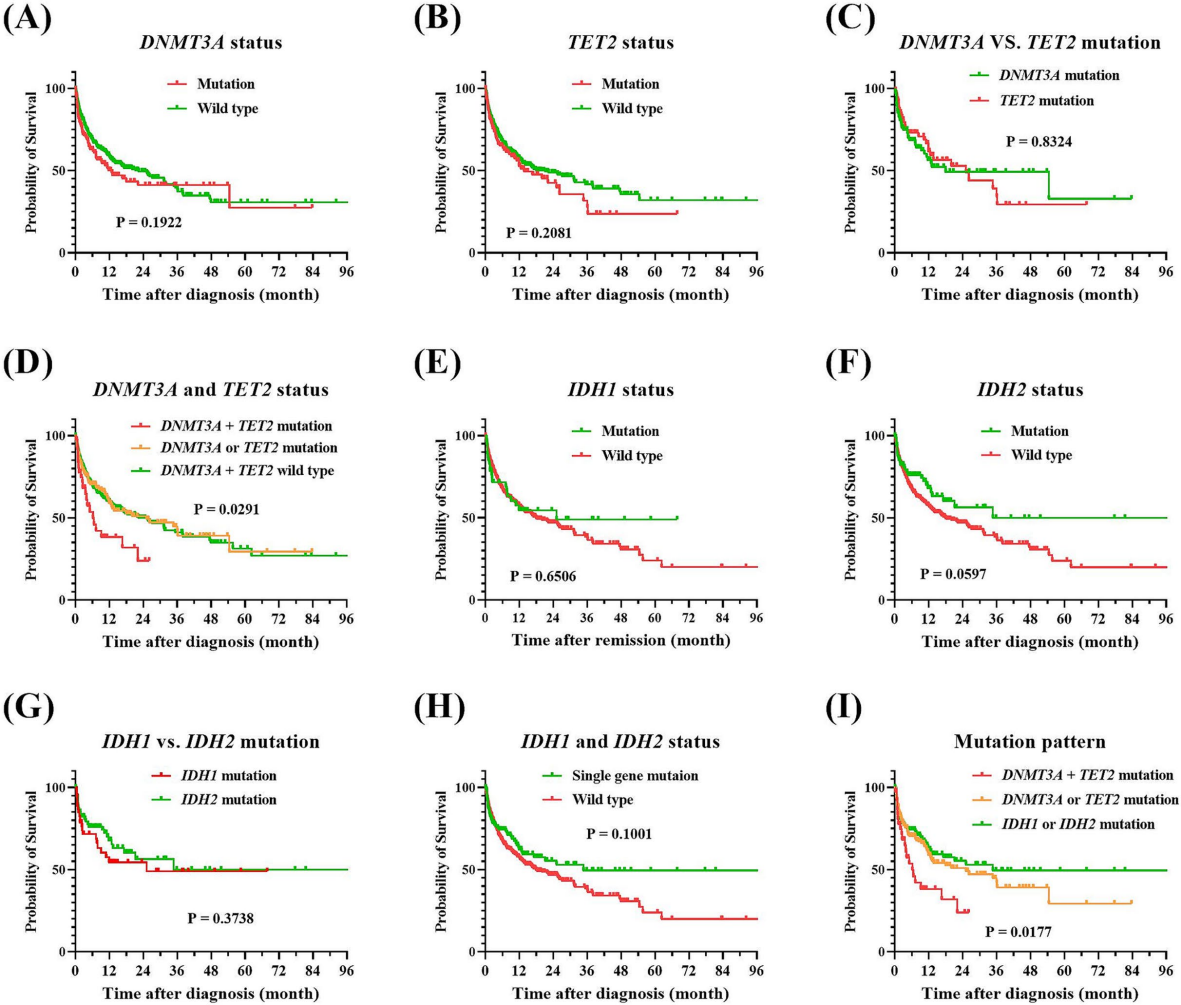
Comparison of overall survival of patients with AML according to their methylation-related gene profiles. **(A)** *DNMT3A* mutation vs. wild-type, *p* = 0.1922. **(B)** *TET2* mutation vs. wild-type, *p* = 0.2081. **(C)** *DNMT3A* vs. *TET2* mutations, *p* = 0.8324. **(D)** *DNMT3A* and *TET2* mutation vs. wild-type, *p* = 0.0291. **(E)** *IDH1* mutation vs. wild-type, *p* = 0.6506. **(F)** *IDH2* mutation vs. wild-type, *p* = 0.0597. **(G)** *IDH1* vs. *IDH2* mutations, *p* = 0.3738. **(H)** *IDH1* and *IDH2* single mutation vs. wild type, *p* = 0.1001. **(I)** Comparative overall survival by methylation-related gene mutations clusters, *p* = 0.0177. Kaplan–Meier analysis was employed to compare overall survival between patients harboring methylation-related gene mutations vs. wild-type counterparts, and the log-rank tests were utilized to assess the differences between the groups. AML, acute myeloid leukemia.

To delve deeper into the impact of demethylating gene mutations on the prognostic outcomes of elderly AML patients, as well as their influence on the therapeutic efficacy of the IC and AZA plus VEN regimen, we performed a univariate prognostic analysis. As they were showed at Figure 3, our findings suggested that mutations in *DNMT3A* and *TET2* were inclined to correlate with an adverse prognosis. Specifically, the median OS for patients with *DNMT3A* mutations, as opposed to those without, were 12.8 months and 22.3 months, respectively, yielding a *p*-value of 0.1922. Similarly, individuals harboring *TET2* mutations experienced a median survival of 13.7 months, in contrast to 21.2 months for their mutation-negative counterparts, with a *p*-value of 0.2081. Moreover, the poorest prognostic outcomes were observed in patients with concurrent *DNMT3A* and *TET2* mutations, exhibiting a median survival of merely 6.2 months and a 2-year overall survival rate of 23.9%. In stark contrast, patients with *IDH1* and *IDH2* mutations exhibited a trend toward improved long-term prognoses. Those with *IDH1* mutations had a median survival of 25.2 months, while the median survival for *IDH2* mutation carriers had not yet been reached, compared to 18.0 months for wild-type patients, with a *p*-value of 0.1709.

**Figure 4:**
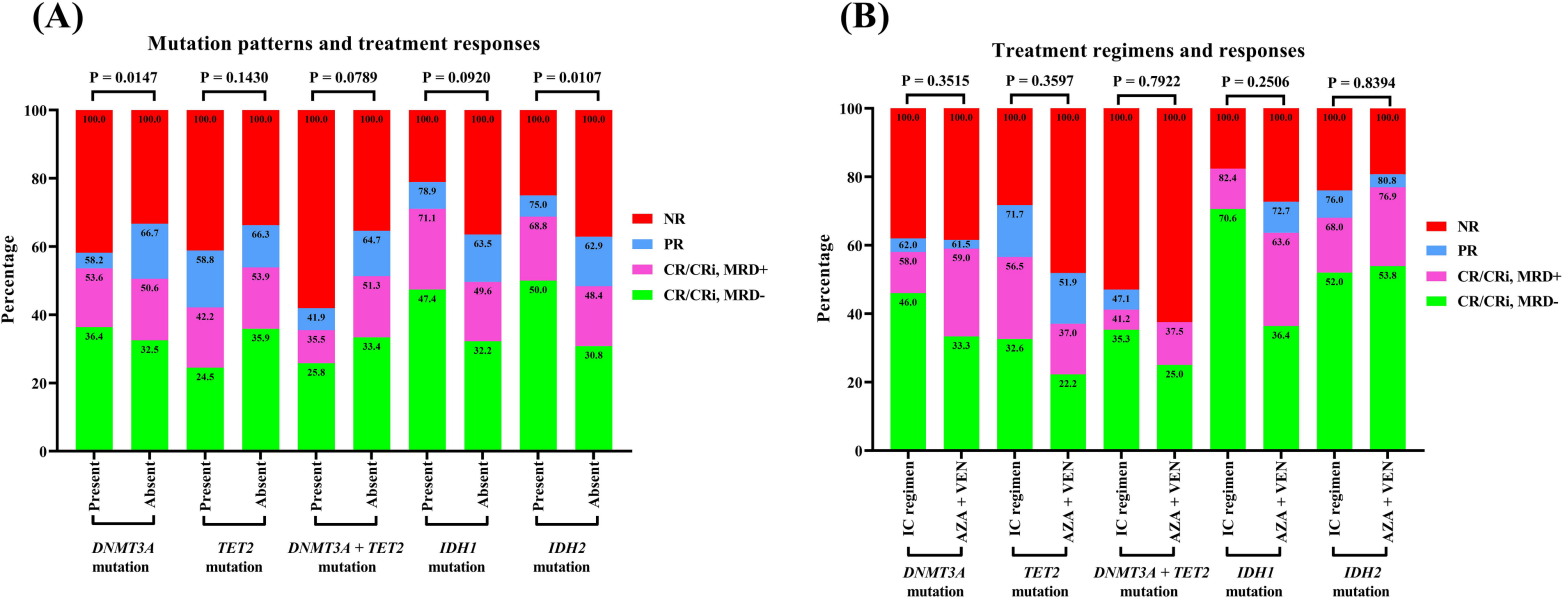
Treatment responses of AML patients with methylation-related gene mutations. **(A)** Mutation patterns and treatment response. **(B)** Treatment regimens and response. A Fisher’s exact test was performed to compare the gene mutation rates between groups. AML, acute myeloid leukemia.

As presented at Figure 4, patients harboring the *DNMT3A* mutation exhibited an induction remission rate that was nearly identical to that of their counterparts without the mutation, with respective complete remission (CR) rates of 53.6% and 50.6%, yielding a *p*-value of 0.5868. The therapeutic efficacy of the IC regimen and the combination of azacitidine with venetoclax were found to be equivalent, with CR rates of 58.0% and 59.0%, respectively, (*p* > 0.9999). Patients with the *TET2* mutation experienced a CR rate of 42.2%, which was lower than the 53.9% observed in non-mutated patients (*p* = 0.0594). Additionally, the induction CR rate in the IC group was 56.5%, surpassing the 37.0% rate in the azacitidine plus venetoclax group, with a *p*-value of 0.1468. Notably, patients who carried both *DNMT3A* and *TET2* mutations had an induction CR rate of merely 35.5%, which below the rates for those with isolated mutations of either DNMT3A or TET2 (*p* = 0.0974). The effectiveness of the IC regimen was similar with the azacitidine plus venetoclax regimen (CR rate: 41.2% vs. 37.5%, *p* > 0.9999). In contrast, patients with *IDH1* or *IDH2* mutations demonstrated a markedly higher induction CR rate compared to wild-type patients, with CR rates reaching 71.1% for *IDH1* mutation carriers and 68.8% for *IDH2* mutation carriers. For *IDH1* mutation carriers treated with the IC regimen, the CR rate was as high as 82.4%, accompanied by a MRD-negative rate of 70.6%. Among *IDH2* mutation carriers, the induction CR rate reached 68.8%, with CR rates of 68.0 and 76.9% for the IC and azacitidine plus venetoclax regimen regimens, respectively (*p* = 0.5414).

## 4 Discussion

Older patients with AML typically exhibit a poor prognosis, with previous studies reporting a 5-year survival rate of less than 10% (2, 6, 7). In a prior study conducted at our center, we detailed the treatment outcomes of elderly AML patients managed before 2016, when conventional chemotherapy was the sole therapeutic option. Among this cohort, patients who received standard IC or low-dose chemotherapy achieved a CR rate of 42.7% during the induction phase. However, the 5-year survival rate for this population was only 13.5%, with a median survival duration of 9.2 months, underscoring the urgent need for more effective therapeutic strategies (22). In contrast, our current data demonstrate that the widespread adoption of novel agents, particularly HMAs and venetoclax, has significantly improved outcomes in elderly AML patients. The overall CR rate increased to 51.3%, the median OS extended to 21.2 months, and the 5-year survival rate reached 30.4%. These findings highlight the substantial advancements in efficacy and prognosis for elderly AML patients in the era of novel therapies compared to the era of traditional chemotherapy alone.

In real-world clinical practice, a patient’s ability to tolerate standard chemotherapy is a key determinant in selecting treatment regimens, such as IC, HMA-based therapy, or the combination of azacitidine and venetoclax. Consequently, in our study, the IC treatment group primarily comprised fit patients, while the HMA- or venetoclax-based treatment groups included older or less fit patients. Our data revealed that 58.7% of patients treated with IC achieved CR, with an ORR of 68.3%, and exhibited superior long-term survival compared to other regimens. This suggests that IC remains the preferred option for fit patients. Conversely, the combination of azacitidine and venetoclax is currently recommended as the first-line regimen for patients unsuitable for IC or those with adverse molecular cytogenetic profiles. The efficacy of this regimen has been validated in numerous clinical trials (23–25). Our study further demonstrated that treatment responses, including CR rates and MRD-negative rates, were comparable between the IC and azacitidine + venetoclax groups and significantly higher than those of other treatment options.

Cytogenetic abnormalities play a pivotal role in risk stratification for AML and are a critical factor in guiding treatment decisions, including the incorporation of targeted therapies (26–29). Methylation-related gene mutations are highly prevalent in elderly AML patients (30). However, the impact of these mutations on prognosis and whether HMAs confer additional benefits in this population remain unclear. In our study, patients with *DNMT3A* mutations exhibited similar initial induction remission rates compared to wild-type patients but had poorer long-term outcomes. In contrast, patients with *TET2* mutations showed both lower induction response rates and worse long-term survival than wild-type patients. Notably, we observed that concurrent mutations in *DNMT3A* and *TET2* were associated with markedly inferior treatment responses and prognosis, a finding not previously reported. On the other hand, patients with *IDH1* and *IDH2* mutations demonstrated significantly better treatment responses and prognosis compared to wild-type patients.

Furthermore, we compared the efficacy of treatment regimens—IC vs. azacitidine combined with venetoclax—in patients with methylation-related gene mutations. Our analysis revealed that patients harboring *DNMT3A* or *IDH2* mutations achieved comparable remission rates with either the IC regimen or the azacitidine + venetoclax combination. In contrast, patients with *TET2* or *IDH1* mutations exhibited higher remission rates, including CR and MRD-negative rates, when treated with the IC regimen compared to the azacitidine + venetoclax regimen. Notably, for patients with concurrent *DNMT3A* and *TET2* mutations, the overall treatment response rate was below 50% regardless of the treatment regimen, although the IC group demonstrated a slightly higher response rate than the azacitidine plus venetoclax group. These findings suggest that treatment regimens incorporating HMAs did not provide additional benefits for patients with methylation-related gene mutations. In other words, the presence of these mutations does not necessitate special consideration when choosing between the IC regimen and a combination regimen incorporating hypomethylating agents (HMAs) and venetoclax.

This study has several limitations. Firstly, as a single-center, retrospective design and extended observation period, some degree of information bias was unavoidable. Secondly, the heterogeneity in the number of treatment cycles across patients should be taken into account when interpreting the results. Thirdly, given the limited sample size and the heterogeneity of testing methods stemming from the extended duration of data collection, we were unable to carry out further subgroup analysis according to the mutation loci of each methylation-related gene mutation. Therefore, further prospective studies are warranted to validate and refine our findings.

## 5 Conclusion

Methylation-related gene mutations are commonly observed in older AML patients. Patients with co-occurring *DNMT3A* and *TET2* mutations showed notably poor treatment responses and survival outcomes. These common methylation-related gene mutations do not significantly influence the choice between IC and azacitidine plus venetoclax regimen.

## Data Availability

The original contributions presented in the study are included in the article/Supplementary material, further inquiries can be directed to the corresponding authors.
