# Supplementary material for: Prognostic impact of methylation-related gene mutations in elderly acute myeloid leukemia: a real-world retrospective analysis
Source: Front Med (Lausanne). 2025 May 13;12:1594784. doi: 10.3389/fmed.2025.1594784 (PMC12106313; doi:10.3389/fmed.2025.1594784)
Supplement: Supplementary file 1 [file Table_1.DOC]

| Supplementary Table 1: Details of Initial Treatment Protocols Employed in This Study. | | |
| --- | --- | --- |
| Regimen | Number | Drugs |
| IC | 240 |  |
| IA protocol | 217 | 4-week cycle: idarubicin at a dose of 8 to 10 mg/m² was administered intravenously once daily for a total of 3 consecutive days; cytarabine at a dose of 100 mg/m² was given by intravenous infusion every 12 hours, continuing for 7 consecutive days. |
| DA protocol | 21 | 4-week cycle: daunorubicin at a dose of 45 to 60 mg/m² was administered intravenously once daily for a total of 3 consecutive days; cytarabine at a dose of 100 mg/m² was given by intravenous infusion every 12 hours, continuing for 7 consecutive days. |
| HA protocol | 2 | 4-week cycle: homoharringtonine at a dose of 2 mg/m², with a maximum dose of 4 mg, was administered intravenously once daily for a consecutive period of 5 to 7 days; cytarabine at a dose of 100 mg/m² was given by intravenous infusion every 12 hours, continuing for 7 consecutive days. |
| HMA monotherapy | 43 |  |
| AZA | 25 | Azacitidine at a dose of 75 mg/m² was administered subcutaneously once daily for a consecutive period of 7 days. |
| DEC | 18 | Decitabine at a dose of 20-25 mg/m² was administered intravenously once daily for a consecutive period of 5 days. |
| HMAs + low-dose chemotherapy | 91 | 4-week cycle: decitabine, 15-20 mg/m2, intravenous injection once a day from days 1 to 5 or azacitidine, 75 mg/m2, subcutaneous injection once a day from days 1 to 7; aclacinomycin, 10 mg per day, intravenous injection once a day from days 3 to 6 or homoharringtonine at a dose of 1.4 mg/m², with a maximum dose of 2 mg, intravenous injection once a day from days 4 to 8; cytarabine: 10 mg/m2, intravenous injection twice a day from days 4 to 10; G-CSF: 5 μg/kg, subcutaneous injection once a day from day 3 to granulocyte deficiency recovery. G-CSF would not be administer when WBC count ≥20×10^9^/L. |
| AZA + VEN | 183 | 4-week cycle: azacitidine: 75 mg/m2, subcutaneous injection once a day from days 1 to 7; venetoclax: 100 mg day 1, 200 mg day 2, 400 mg from days 3 to 28, oral administration once a day. When severe hematological toxicity or complications occur, the dose of venetoclax might be adjusted downward or temporarily discontinued. |
|  |  | For patients who were intolerant to venetoclax or experience significant hematological toxicity, the dosing may be adjusted to 200 or 300 mg per day, and venetoclax was temporarily discontinued in patients who developed neutropenia with severe infection. |
| Other protocols | 28 | The treatment regimens included low-dose chemotherapy, venetoclax in combination with chidamideine or cytarabine. |
| IC: intensive chemotherapy; HMA: hypomethylating agents; AZA: azacitidine; DEC: decitabine; VEN: venetoclax. Chemotherapy cycles were adjusted according to the patient's bone marrow hematopoietic recovery and the occurrence of concurrent complications. For patients with FLT3 mutations, FLT3 inhibitors such as sorafenib and gilteritinib were incorporated into the aforementioned treatment regimens. | | |
