# Supplementary material for: Prognostic impact of methylation-related gene mutations in elderly acute myeloid leukemia: a real-world retrospective analysis
Source: Front Med (Lausanne). 2025 May 13;12:1594784. doi: 10.3389/fmed.2025.1594784 (PMC12106313; doi:10.3389/fmed.2025.1594784)
Supplement: Supplementary file 2 [file Table_2.DOC]

| Supplementary Table 2. Specific Gene Regions Analyzed for Mutation Detection in This Study. | | |
| --- | --- | --- |
| Genes | Detection regions | Hotspot mutation sites |
| *DNMT3A* | All exons of the gene | R882 |
| *TET2* | All exons of the gene |  |
| *IDH1* | Exon4 | R132 |
| *IDH2* | Exon4 | R140, R172 |
